# Integrating Historical Person Registers as Linked Open Data in the WarSampo Knowledge Graph

**DOI:** 10.1007/978-3-030-59833-4_8

**Published:** 2020-10-27

**Authors:** Mikko Koho, Petri Leskinen, Eero Hyvönen

**Affiliations:** 8 Linköping University, Linköping, Sweden; 9 University of Amsterdam, Amsterdam, Noord-Holland The Netherlands; 10 Department of Computer Science, Vrije Universiteit Amsterdam, Amsterdam, Noord-Holland The Netherlands; 11 St. Pölten University of Applied Sciences, St. Pölten, Austria; 12 FIZ Karlsruhe – Leibniz Institute for, Karlsruhe, Germany; 13 Karlsruhe Institute of Technology, Karlsruhe, Germany; 14 UAS St. Pölten, St. Pölten, Niederösterreich Austria; 15 Vienna University of Economics and Business, Vienna, Wien Austria; 16 VU Amsterdam, Amsterdam, The Netherlands; 17 ADAPT Centre, Trinity College Dublin, Dublin, Ireland; 18 Semantic Computing Research Group (SeCo), Aalto University, Espoo, Finland; 19 HELDIG – Helsinki Centre for Digital Humanities, University of Helsinki, Helsinki, Finland

## Abstract

Semantic data integration from heterogeneous, distributed data silos enables Digital Humanities research and application development employing a larger, mutually enriched and interlinked knowledge graph. However, data integration is challenging, involving aligning the data models and reconciling the concepts and named entities, such as persons and places. This paper presents a record linkage process to reconcile person references in different military historical person registers with structured metadata. The information about persons is aggregated into a single knowledge graph. The process was applied to reconcile three person registers of the popular semantic portal “WarSampo – Finnish World War 2 on the Semantic Web”. The registers contain detailed information about some 100 000 people and are individually maintained by domain experts. Thus, the integration process needs to be automatic and adaptable to changes in the registers. An evaluation of the record linkage results is promising and provides some insight into military person register reconciliation in general.

## Introduction

A way to enhance our understanding about history is to integrate data from complementary information sources in an interoperable way. In *record linkage (RL)* [2, 6, 13], the goal is to find matching structured data records between heterogeneous databases. A typical application scenario is matching person records in different person registers, which contain structured data about some same persons, but are expressed using different metadata schemas and notations. Using RL, richer global descriptions of persons can be created based on local datasets.

This paper concerns the problem of entity reconciliation and RL of persons in military historical person registers. As a case study, three complementary datasets about some 100 000 Finnish Second World War soldiers in WarSampo [7, 10] are considered. A probabilistic record linkage [6] solution for linking person records is presented, as well as promising evaluation results. The key idea is to assign weights to various comparisons of metadata fields between person registers. The weights can tell us what information is important for disambiguating person records in the military history context.

After the matches between registers are generated, information is aggregated into the actor ontology, which contains the identities and enriched metadata of each person. Integrating the person registers into a single *knowledge graph (KG)* facilitates biographical and prosopographical research [9].

The WarSampo KG is published as open data^1^ and is part of the international Linked Open Data Cloud. The WarSampo portal^2^ [7] demonstrates the usefulness of the resulting KG integrated from various sources. WarSampo uses Linked Data and the event-based *CIDOC Conceptual Reference Model (CRM)*^3^ together as a basis for harmonizing various datasets about Finland in the Second World War. The portal provides nine customized interactive “perspectives” on the data: (war) Events, Persons, Army Units, Places, Magazine Articles, Casualties, Photographs, War Cemeteries, and Prisoners of War. Since its opening in 2015, the WarSampo portal has been used by more than 710 000 end users, corresponding to more than 10% of the population in Finland.

**Related Work.** Overviews of the RL field are presented in [6, 13]. Antolie et al. [1] present a case study of integrating Canadian World War I data from three sources: one of soldiers, one of casualties, and a census dataset, using a series of handcrafted deterministic RL processes. Research use of the resulting longitudinal data is demonstrated. Cunningham [3] presents integrating a World War I veteran military service record with a census database using a deterministic RL process and provides findings of quantitative analysis of the data for historical research.

The Historical Population Register (HPR) of Norway is pursuing to cover the country’s whole population in 1800–1964 combining information with RL from church records and censuses [12]. The Links project^4^ has similar goals in the Netherlands aiming to reconstruct all nineteenth and early twentieth century families in the Netherlands based on all civil certificates from this period.

## Data: WarSampo Person Registers

In WarSampo, information about a single person can be found in multiple person registers, each bringing in some new information about the person. The information found from multiple sources can be combined to create a more complete biography of the person. However, it is challenging to reliably say whether two similar looking person records in different registers refer to the same actual person as they contain no common fields with shared unique values.

The military rank and military unit of a soldier are prone to change in time due to promotions or even demotions. There can be different spellings of a name, middle names can be missing, and in Finland many originally foreign surnames were translated into Finnish in the early 20th century. In practise, the same full name can refer to different persons, and different names can refer to the same person. There are currently three different person registers in WarSampo:

- **1. Initial Actor Ontology. ** The ontology containing 5600 people, and also military units, has been created from various data sources which provide varying levels of detail [11]. For most of the people there is rich biographical metadata, e.g. a person’s full name, the dates and places of birth and death, occupation, and dates of promotions during the military career. However, in some cases the level of detail is not sufficient for disambiguation, e.g., only a surname and military rank may be known.
- **2. Register of Military Death in the Finnish Wars 1939–1945. ** The register contains 94 700 death records (DR) [8], depicting the status of the person at the time of his/her death. The spreadsheet source data contains detailed information about the known Finnish persons who perished in WW2. There are 32 columns of structured information about each person, with each cell having a single literal value.
- **3. Register of the Prisoners of War in Soviet Union 1939–1945. ** The register contains 4200 prisoner records (PR) [9], depicting the status of persons at the time when they were captured. It was published in WarSampo on November 2019. The spreadsheet source data contains mostly very detailed information about each known Finnish prisoner of war. The spreadsheet contains 45 columns of information about each person, gathered from, e.g., various archives. Often a single cell contains multiple values corresponding to information in different sources, following a pre-defined cell formatting. Most of the cells contain well-formed literal values, like the municipality of birth, military rank, and date of returning from captivity.

## Method: Linking Person Records

The WarSampo KG is built from source datasets using a repeatable data transformation pipeline [10]. In this approach, the domain experts maintain the primary data in the original native format, i.e., typically spreadsheets. When a source dataset is updated, the pipeline can be used to easily recreate the whole KG with the updated data.

The pipeline transforms the source spreadsheets of DRs and PRs into RDF, mapping the columns to RDF properties, with possibly multiple values per property. Automatic probabilistic entity linking processes then link the records to the WarSampo domain ontologies of military ranks, units, occupations, people, and places. This *semantic reconciliation* improves the interoperability [4] of the person registers. If the related domain ontologies are updated, the whole integration process can be redone to account for the changes in the probabilistic entity linking.

The person record linkage is performed after linking the metadata values to domain ontologies. This is challenging because of heterogeneity of the metadata schemas, ambiguous metadata annotations, temporal changes, and errors in the data. Approximate similarity matches of metadata fields is often useful when working with noisy historical person records [1].

The two record linkage scenarios that are needed to tackle for integrating data from all three person registers are:

- **RL1.** DRs (94 700 person records) linked with the initial actor ontology (5600 persons)
- **RL2.** PRs (4200 person records) linked with the actor ontology enriched with the DRs (99 667 persons)

The first developed solution, applied in both scenarios, was a deterministic (or rule-based) RL, in which all person pairs were compared with each other, and scored based on a pre-defined handcrafted formula. This was manually evaluated to provide at least satisfactory results (precision estimated to be at least 0.9), but as the datasets were being updated and the ontologies evolving, manually maintaining the scoring formula was decided to be not sustainable.

The second solution is to use probabilistic RL [6], with a logistic regression-based machine learning implementation employing the Dedupe Python library [5]. Results from the previous solution are used as training data, consisting of 216 matches for *RL1* and 1234 matches for *RL2*. Of these, the ones close to the match acceptance threshold were manually validated to be correct. Person instances or person records with only 3 or less metadata fields for the RL are ignored as too ambiguous in the linking process. The RL solution^5^ is open-source, and is used in the transformation processes of the DRs^6^ and the PRs^7^. A run of the probabilistic RL process completes within a few hours in both of the scenarios on an average desktop computer.

The scoring of possible pairs between the PRs and the persons already integrated to WarSampo, i.e., initial actor ontology and DRs, are performed using the comparisons of properties shown in Table 1. The weighted sum of the individual comparisons is used as a confidence that a given pair of records is a match, i.e., that it refers to the same real world person. If the weighted sum is above a given, manually fine-tuned threshold, the records are considered a match. The comparisons of type *string* use hyper-parameter optimization to find the best performing string comparison for the values, e.g., Jaro-Winkler. The *intersection* comparisons compare the one or more URI values of both records to see if there is a matching URI or not. The *date* comparisons measure the distance of two dates based on CIDOC CRM time-spans, which have separate earliest and latest dates. The *numerical* comparison measures the distance of numerical values.

To address temporal changes in a person’s military rank and the observed variance in the use of different private level ranks, a comparison based on the comparative level of a rank is used. This also addresses the rather permanent separation between enlisted ranks and commissioned officers.

**Table 1.** Used metadata comparisons between the registers for the probabilistic RL.

Property	Comparison type	Binary/Continuous variable
Given names	String	Continuous
Family name	String	Continuous
Municipality of birth	Intersection	Binary
Date of birth [earliest]	Date	Continuous
Date of birth [latest]	Date	Continuous
Date of death [earliest]	Date	Continuous
Date of death [latest]	Date	Continuous
Municipality of death	Intersection	Binary
Activity end	Date	Binary
Military rank	Intersection	Binary
Military rank level	Numerical	Continuous
Military unit	Intersection	Binary
Occupation	Intersection	Binary

**Aggregating Personal Information. ** After the links of records between registers are generated, information is aggregated into the actor ontology, which contains the identities and basic metadata of each person, with a data model based on CIDOC CRM. New person instances are created in the actor ontology for the records that did not match any existing person and existing person instances are enriched with new information. The person records are modeled as instances of CIDOC CRM’s document class, which are linked to the person instances in the actor ontology.

## Results and Evaluation

The record linkage scenario *RL1* results in 620 DRs linked to matching people in the 5611 pre-existing person instances, corresponding to 11% of the people in the actor ontology. For the remaining 94 056 DRs, new person instances are created.

The *RL2* scenario results in 1255 person records linked to matching people in the 99 667 pre-existing person instances, corresponding to 30% of the PRs. Of the matches, 1234 already exist in the training data as the initial deterministic solution was already quite successful in matching the records based on an early version of the prisoner register. For 2945 PRs, new person instances are created in the actor ontology.

**Comparison Weights. ** The learned comparison weights depict what information is useful for disambiguating person records in the military history context. The weights of the comparisons vary a little as new runs on updated data are done, but their general magnitude is stable. For the newest WarSampo data transformation, the comparison weights in the *RL2* scenario in descending importance order are: family name (2.3), municipality of birth (2.0), given names (1.4), date of birth earliest (1.2), birth date latest (1.2), military rank (1.0), occupation (0.9), military unit (0.8), military rank level (0.8), municipality of death (0.4). The remaining comparisons have weight under 0.1.

Names, municipality of birth and date of birth are intuitively very important personal details defining a persons identity. As the date of birth is split into two comparisons, it’s overall importance can be summed up to 2.4, making it the single most important metadata field. The summed weight of military rank, 1.8, is higher than that of given names. Military unit is also important, nearly as much as a person’s occupation. Occupation of soldiers probably have not changed during the war, but what is considered a persons occupation might vary depending on the situation and accountant.

**Linking Quality. ** Due to the mostly rich data of each person contained in the person registers, manual evaluation of found links is usually possible, by examining the data in detail. This enables estimating the RL precision. Recall evaluation however, would need manual inspection of a very high amount of possible pairs, of which some have very little information. Also, the DRs are known to contain plenty of errors. Hence, it is in many cases difficult to confidently determine the true negative results, i.e., the cases where there is no match, which is crucial for the recall evaluation. However, manual inspection of matches that almost met the matching threshold were either ambiguous or false, suggesting that the recall is adequate.

The precision of the record linkage in both scenarios *RL1* and *RL2* was manually evaluated to be 1.00, based on randomly selecting 150 links from the total of 620 links for *RL1*, and 200 links from the total of 1397 links for the *RL2*. The information on the person records and the person instances was compared, and all of the linked records were interpreted to be depicting the same actual persons with high confidence.

**Using the Aggregated Information. ** The aggregation of information from multiple sources provides more full soldier biographies than when using individual sources. For example, the PRs fill a gap that would otherwise exist for each of the captured soldiers by providing, e.g., detailed information about their movements between prison camps.

There are also person related documents that are linked to the person instances or their military units, i.e., a large collection of wartime photographs, hand-written digitized war diaries, and war veteran magazine articles. These easily provide further information for people studying for example the war paths of their relatives.

The Persons perspective of the WarSampo portal uses the aggregated person instances and information directly from the linked person records to create a unified view of all the information of each person, in a sense creating a “homepage” for them.^8^ In addition to showing the aggregated information, links are provided to related documents as well as related military units and people.

## Discussion

This paper presented the probabilistic record linkage process used in WarSampo to integrate heterogeneous person registers into a reconciled KG, which uses training data created by a simpler deterministic RL solution. The solution is capable of automatically handling updates in the person registers or related domain ontologies. The aggregated information can be used for, e.g., biographical or prosopographical research by historians, or for study and exploration by interested citizens.

The weights of different metadata field comparisons, assigned using logistic regression, shed light on what metadata fields are useful in disambiguating person references in the military history context. Military rank and military unit are both important person details when determining the identity of a person depicted in a person record.

The data is published openly on SPARQL endpoint and on the WarSampo portal, where anyone can evaluate the links between different person records as they are modeled as separate resources in the data and information sources are shown to users. The Persons perspective of the portal displays all information about a single person in the KG. The Casualties and Prisoners perspectives provide faceted search and visualizations to explore, study, and analyze the DRs and PRs, respectively. In the future, a similar perspective for the aggregated person instances would be useful, where a user can conduct similar prosopographical analysis over all the persons.

The solution is scalable and can be further used to integrate more person registers into WarSampo. For considerably larger person registers, a blocking strategy [2] based on the metadata values should be adopted to reduce the number of comparisons. The presented approach is applicable also to other studies integrating historical person registers. A simple deterministic RL process can be useful for creating training data for a probabilistic RL process in similar scenarios where the process needs to be able to handle regular data updates automatically.

In the future, a register of the soldiers who survived the war would be a valuable addition to WarSampo, providing the means to study subjects such as what affects the soldiers’ likelihood of surviving the war.

## Footnotes

1 https://doi.org/10.5281/zenodo.3431121.

2 http://sotasampo.fi/en/.

3 http://cidoc-crm.org.

4 Cf. the project homepage https://iisg.amsterdam/en/hsn/projects/links and research papers at https://iisg.amsterdam/en/hsn/projects/links/links-publications.

5 https://github.com/SemanticComputing/warsa-linkers.

6 https://github.com/SemanticComputing/Casualty-linking.

7 https://github.com/SemanticComputing/WarPrisoners.

8 Cf. an example person “homepage” at https://www.sotasampo.fi/en/persons/person_65.
